# Determination of feed value of cherry, apricot and almond tree leaves in ruminant using *in situ* method

**Published:** 2012-09-21

**Authors:** M.K. Nahand, R.S. Doust-Nobar, N. Maheri-Sis, S. Mahmoudi

**Affiliations:** *Department of Animal Science, Shabestar Branch, Islamic Azad University, Shabestar, Iran*

**Keywords:** Dry matter degradability, *In situ*, Ruminant nutrition, Tree leaves

## Abstract

In the present study, chemical composition and *in situ* rumen dry matter degradability (DMD) of some tree species (cherry, apricot and almond tree leaves) were determined. Crude protein (CP) concentration varied from 6.76% for almond tree to 2.76% for cherry tree, neutral detergent fiber (NDF) and acid detergent fiber (ADF), from 29.2, 20.8% for apricot tree to 20.8 and 15.8% for almond tree leaves respectively. Polyphenol and tannin composition measured from 3.49, 1.2% for almond tree to 1.51 and 0.61% for apricot tree, respectively. *In situ* rumen degradability was carried out in three fistulaed Taleshi native male cattle which were incubated at times of 0, 4, 8, 16, 24, 48, 72 and 96-hour. Almond leaves had higher potential degradation (a+b) for dry matter (92.37%) and cherry leaves showed lower potential degradation (84.12%), respectively. Effective rumen degradable dry matter at rate of 0.05/h varied from 69.86% for almond tree to 52.20% for cherry leaves. Results showed that the almond leaves were higher in nutritive value than cherry and apricot leaves. Therefore, almond tree leaves could be used with forage in ruminant diets to reduce cost of animals feed requirements. Overall, it seemed that the tree leaves used in this study, had a higher nutritive value in ruminant’s nutrition, however more experiments are needed for an accurate determination of nutritional values of these resources.

## Introduction

According to the type and concentration of compounds in the diet, the effects can be favourable or unfavourable on intake and digestive parameters. Reported associative effects between forages show a large variability among studies. This reflects the complexity and multiplicity of nutritional situations affecting intake and the rumen function in a given animal (Niderkorn and Baumont, 2009). There is a great need to feed ruminants with balanced feed to improve their production of meat and milk. Several tree species could be effective sources of providing fodder nutrition during normal as well as scarcity periods (Reddy, 2006). Although there are many advantages of the forages and leguminous crops over tree crops, the leaves of certain trees can be as nutritious as those of fodder legumes (Soliva *et al.*, 2005). The presence of legume forages and tree forages in pastures have been generally accepted to improve ruminant productivity in both temperate (Ulyatt, 1980) and tropical pastures (Milford, 1967).

Fodders trees may be used to feed ruminants where high protein feed resources are scarce or unavailable. They should be planted where they have advantages over more conventional forage crops. In general, it is not economic to grow trees as a high biomass crop to provide a basal diet for ruminants. Supplements of tree foliage can increase growth rates of cattle above that of a supplement of urea. This has been shown in studies where tree foliage has been fed with ammoniated straw which could be anticipated to oversupply rumen ammonia on these diets.

However, it does not rule out the correction of other deficiencies by minerals in the tree foliage, such as sulphur (FAO, 1997). Recently, trees and shrubs have been introduced into cropping and grazing systems to provide green fodder high in protein to supplement the available low protein forage.

These are grown in banks or hedges, between crops (alley farming) or as components of pastures and also as shade trees. The four potential roles of tree foliage in ruminant nutrition are as a (FAO, 1997): (1) High quality, high digestibility biomass resource. (2) Supplement to provide nutrients deficient in the diet, an enhancement of microbial growth and digestion of cellulosic biomass in the rumen of cattle, sheep and goats. (3) Source of protein that escapes rumen degradation to be digested in the intestines and enhance the protein status of the animal. (4) Source of vitamins and minerals to complement deficiencies in the basal feed resource.

Researches that were conducted for evaluation of nutritional value of tree leaves shown that tree leaves have potential nutritive value in ruminants nutrition (Aganga and Tshwenyane, 2003; Kamalak *et al.*, 2004; Azim *et al.*, 2011). Chumpawadee and Pimpa (2009) reported that leaves of fodder trees should be used as fiber sources in Total Mixture Ration (TMR) because of their high content of protein, minerals and vitamins and availability in the dry season. In addition, the toxic substance in leaves can be reduced by sun drying.

In arid areas where the growth of herbaceous plants is limited by lack of moisture, leaves and edible twigs of trees and shrubs can constitute well over 50% of the biomass production of range-land. At high altitudes, tree foliage may provide over 50% of the feed available to ruminants in the dry season, branches being harvested and carried to the animals. Even in regions of higher rainfall where grass supplies the major proportion of the dry matter eaten by ruminants, tree leaves and fruits can form an important constituent of the diet, particularly for small ruminants.

In this regard, goats may be able to utilize tree leaves as no sign of toxicity was found in goats consuming 10-23 g kg^-1^ of tannins-rich leaves (Phale and Madibela, 2006). Chemical composition, *in situ* degradation and *in vitro* digestibility can be considered useful indicators for the preliminary evaluation of the likely nutritive value of previously uninvestigated shrubs (Nahand *et al.*, 2011). Semi-arid browses are forages with high protein concentration and effective *in vitro* DM digestibility.

Several methods such as *in vivo*, *in situ* and *in vitro* techniques have been used in order to evaluate the nutritive value of feedstuffs (Kamalak *et al.*, 2004; Maheri-Sis *et al.*, 2007a, 2007b; Nahand *et al.*, 2011). Information on the nutritional value of tree leaves for ruminants especially in Iran is limited. Therefore, the objective of the present study is to assess the nutritional composition of tree leaves by its chemical composition, *in situ* DMD and *in situ* digestibility kinetics.

## Materials and Methods

This study was conducted over the period from September 2009 to March 2010 at Islamic Azad University, Shabestar branch, Department of Animal Science.

### Sampling leaves

Tree Leaves were collected from the trees species during the fall seasons (approximately 5 kg of each sample). Green leaves plus their petiole were picked randomly by hand from different specimens per species at a site, and pooled. After collection leave samples were dried for one week. Approximately 500 g dry samples were put in a paper bag and taken to the laboratory for chemical analysis and *in situ* technique measurements. The species of experimental sample for almond, cherry and apricot tree leaves were, *Prunus amyydalus, Prunus avium*, and *Prunus armanica*, respectively.

### Animals

Three fistulated Taleshi male cattle (5 years old, about 550 kg live weight) were used for the application of nylon-bag techniques. Animals fed twice daily with a diet containing alfalfa hay (60%) and concentrate (40%). The concentrate mixture contained 55% barley grain, 15% soybean meal, 5.7% cotton seed meal, 21% wheat bran, 0.3% salt, 1% di calcium phosphate (DCP), 1% calcium carbonate and 1% vitamin- mineral premix. Also water was available *ad libitum* during the experiment period.

### Chemical analysis

CP, Ether Extract (EE) and ash contents of the tree leaves were determined according to AOAC (1990). CP analysis was by the process of Kjeldahl method (AOAC, 1990). It was performed by breaking down of 2 g sample (n=2) in 25 ml concentrated H_2_SO_4_ plus selenium, using Gerhardt Kjeldahtherm (Gerdart GmbH + Co. KGFabrikfur Laborgerate Postfach 1628 D53006 Bonn) until an opaque colour was obtained. The digested sample was rested for 12 h, diluted with distilled water and made up to 250 ml in a volumetric flask. Five ml of the digest was taken and distilled with 40% NaOH and the ionised ammonium was trapped by boric acid. The distillate was immediately titrated (n=3) with 0.01N HCl. In order to obtain the percentage CP, the amount of N was multiplied with factor 6.25. NDF and ADF were determined as reported by Van Soest *et al*. (1991). Non fibrous carbohydrates (NFC) were determined by difference, NFC =100 – (NDF + CP + EE + Ash) (NRC, 2001). Total phenolic and tannin composition were determined by butanol-HCl method as described by Makkar *et al*. (1995). All chemical analyses were carried out in triplicate (Hagerman, 1995).

### In situ nylon bag technique: Ørskov and McDonald, (1979).

*In situ* technique was used in this study. However, this is identical to the *in sacco* technique or the Terylene (Dacron) - or nylon-bag techniques. The procedure was as follows:

Samples of dried and milled feed (to pass a 3 mm screen) or wet minced samples were placed in nylon bags (usually 16×8 cm, pore size 45 to 60 μm). About 2 to 5 g, depending on density, were weighted precisely into each bag. The tied-up bags were incubated in the rumen of sheep or cattle on an appropriate diet by suspending them from a rumen cannula. They were then withdrawn after various intervals of time, washed and dried. Degradability of dry matter, nitrogen, energy, etc., can thus be measured against time.

The bags were incubated in the rumen of the three fistulated cattle. Nylon bags were withdrawn at 0, 4, 8, 16, 24, 48, 72 and 96 h after insertion. For soluble fraction (0 h) measurement was obtained by soaking the two bags of sample in warm water (37°C) (for 1 h). The 0 h and incubated bags were then washed with cold water for 15 min in a washing machine and dried for 48 h at 60°C. The DM degradation data were fitted to the exponential equation p=a+b (1-e^-ct^) (Ørskov and McDonald, 1979; Ørskov *et al.*, 1980). To determine the degradation characteristics (a, b, a+b, c and ED); where p is the DM degradation at time t; a, denotes washing loss (representing the soluble fraction of the feed); b, insoluble fraction; c, is the rate of degradation of fraction b; and ED, denotes effective degradability (ED), calculated at an outflow rate of (0.02, 0.05 and 0.08 h^-1^).

After incubation, DMD for each bag, for each incubation period and for each cattle was calculated separately with formulas suggested by Ørskov and McDonald (1979) using FITCURVE software version 6 (Chen, 1995).

p=a+b (1-e-^ct^)

Where:

P: is the DM degradation at time t

a: denotes washing loss (representing the soluble fraction of the feed)

b: insoluble fraction

c: is the rate of degradation of fraction b

e = 2.7182 (Natural logarithm base)

Following determination of these parameters, the ED of DM in tree leaves was calculated using equation described by Ørskov and McDonald (1979): ED = a + (b*c)/(c + k)

Where:

ED = Effective degradability for response variables (%)

a = Highly soluble and readily degradable fraction (%)

b = Insoluble and slowly degradable fraction (%)

c = Rate constant for degradation (h^-1^)

k = Rate constant of passage (h^-1^)

When calculating ED, rate of constant passage was assumed to be 0.02, 0.05 and 0.08 per hour (Bhargava and Ørskov, 1987) so that the results could be extrapolated to other ruminants that differ in rumen capacity.

### Statistical analysis

All of data were analyzed by Statistical Analyze Software (SAS) (1999). ANOVA was carried out for the comparison of *in situ* kinetics and dry matter degradation value. Significance between individual means was identified using Duncan’s multiple range Test. Mean differences were considered significant at *P* < 0.05. Standard errors of means were calculated from the residual mean square in the analysis of variance.

## Results

The chemical composition of almond, cherry and apricot tree leaves are given in Table 1. The NDF and ADF in almond tree were lower than that of other species, while CP, Polyphenolic compounds were higher than that of cherry and apricot tree leaves. The tannin content of the leaves was low, ranging between 0.06 to 1.206 % DM. Although no statistical analysis of species composition was carried out, there was a considerable variation between species in terms of chemical composition.

**Table 1:** The chemical composition of leaves (%).

Treatments	Almond tree	Cherry tree	Apricot tree
Dry matter	91.32	91.11	93.11
Ether extract	3.5	8	6
Crude protein	6.76	2.76	3.54
Neutral detergent fiber	20.8	27.6	29.2
Acid detergent fiber	15.8	20	20.8
Ash	8.80	9.30	15
Polyphenolic compounds	3.49	3.44	1.51
Condensed tannin	1.2	2.18	0.617
Nonfibrous carbohydrates	60.14	52.34	46.26

## Discussion

The variation in chemical composition among these species may be partly due to the genotypic factors and some of environmental factors that control accumulation of forage nutrients (Minson, 1990; Chumpawadee, 2009).

The tannin values in tree leaves could be higher than the values obtained in this study, since a considerable amount of tannins are bound to either fiber and/or proteins and remain unrestricted (Jackson *et al.*, 1996). The beneficial effect of feedstuffs containing low levels of tannins could be due to the protection of proteins from microbial degradation by tannins, thus increasing the amount of undegraded protein entering the small intestine (Barry *et al.*, 1986). In addition, a higher flow of microbial protein to the intestine has been observed as a result of higher efficiency of microbial protein synthesis (Getachew *et al.*, 2000). However, higher concentration of tannins in the diet is associated with reduction in organic matter digestibility (Silanikove *et al.*, 1997).

**Fig. 1:**
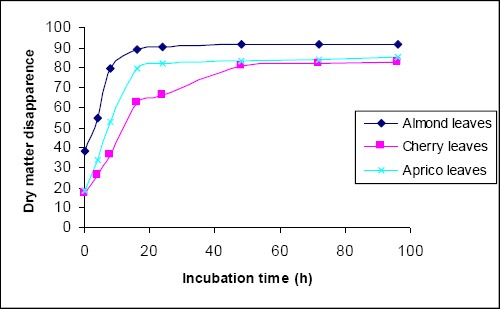
DM degradation of tree leaves.

DM disappearance of tree leaves from bags at different rumen incubation times is given in Figure 1 and the estimated degradation kinetics is presented in Table 3. There was an increase in DM disappearance associated with the increasing time of incubation. At all incubation times DM disappearances for almond ED of the examined nutrient components were calculated using the outflow rates of 0.02, 0.05 and 0.08/hr. ED decreased by increasing out flow rate (In case of maintenance level feeding (Out flow rate 0.02 h^-1^)). ED of DM, were 81.10%, for almond leaves and 66.46% for cherry leaves, respectively.

**Table 2:** *In situ* degradability of leaves (%).

Incubation times	Almond	Apricot	Cherry	*P* value	SEM
0	27.24^a^	18.21^b^	17.48^b^	0.001	0.311
4	40.76	33.93	26.37	0.079	3.6
8	59.72^a^	52.69^ab^	36.01^b^	0.0213	4.35
16	86.52^a^	79.87^a^	62.30^b^	0.004	2
24	88.24^a^	82.35^a^	66.34^b^	0.007	3.19
48	90.76^a^	83.59^ab^	80.90^b^	0.014	1.65
72	90.71^a^	84.24^b^	82.33^b^	0.001	0.52
96	91.16^a^	85.06^b^	82.86^c^	0.008	0.184

**Table 3:** *In situ* degradability and effective degradability of tree leaves (%).

Estimated Parameters	Almond	Apricot	Cherry	*P* value	SEM
a	23.52^a^	14.78^b^	14.32^b^	0.0008	0.94
b	68.84	71.11	69.79	0.436	1.16
a+b	92.37^a^	85.90^b^	84.12^b^	0.0001	0.008
c	0.104^a^	0.107^a^	0.060^b^	0.0108	0.166
Lag time	0.542	0.454	0.782	0.352	0.015
Effective degradability					
0.02	81.10^a^	74.76^b^	66.46^c^	0.0001	0.73
0.05	69.86^a^	63.30^b^	52.20^c^	0.0003	1.33
0.08	62.30^a^	55.60^b^	44.13^c^	0.0004	1.49

In 5-8% outflow rates, the ED for almond leaves was significantly higher (P<0.05) than apricot and cherry leaves. Effective DM degradability decreased with the increase in outflow rates in this study. Mupangwa *et al*. (1997) observed ED of DM to decrease as the outflow rate increase.

## Conclusion

The CP concentration of the three leaf species analyzed, varied between approximately 6.7 and 2.7%. It is concluded that the tree leaves from the species in the present study contained low concentrations of CP. Factors such as differences in climate, soil types, etc. could have contributed to these differences.

Results of current study indicated that almonds tree leaves had approximately higher rumen degradation dry mater. As well as soluble fraction (b) and Potential degradability (a+b) than cherry and apricot leaves, simultaneously, according to our results it seems almond leaves had better nutritive value than cherry and apricot leaves as feedstuff for ruminants.

In Conclusion, it seems that the trees leaves have a better nutritive value for ruminant’s nutrition and it could improve animal production and lead to prosper feedstuff in livestock science, but more experiments are needed for accurate determination of nutritional values of these resource. The rumen bag technique has been used for many years to study the degradation of forages, however, the digestibility of a feedstuff is defined by the potential degradability of the material, the rate of degradation of this potentially degradable fraction and its residence time in the rumen (i.e. its effective degradation) plus digestion after the rumen fermentation in the hind gut. As such, they have potential as forage for farmers during the long period of dry season when feed is scarce. Our results claim that it is possible to use fruit’s leaves as animal feedstuff in poor agro-products areas.
